# AFM dendritips functionalized with molecular probes specific to cell wall polysaccharides as a tool to investigate cell surface structure and organization

**DOI:** 10.1016/j.tcsw.2019.100027

**Published:** 2019-08-05

**Authors:** Marion Schiavone, Nathalie Sieczkowski, Mathieu Castex, Emmanuelle Trevisiol, Etienne Dague, Jean Marie François

**Affiliations:** a LISBP, UMR INSA-CNRS 5504 & INRA 792, F-31077 Toulouse, France; b Lallemand SAS, 19, rue des briquetiers, 31702 Blagnac, France; c CNRS, LAAS, 7 avenue du colonel Roche, F-31400 Toulouse, France

**Keywords:** Cell surface, Cell wall, β-Glucan, Chitin, AFM dendrtips, Anti β-1, 3-glucan antibodies, Molecular interaction

## Abstract

The yeast cell wall is composed of mannoproteins, β-1,3/β-1, 6-glucans and chitin. Each of these components has technological properties that are relevant for industrial and medical applications. To address issues related to cell wall structure and alteration in response to stress or conditioning processes, AFM dendritips were functionalized with biomolecules that are specific for each of the wall components, which was wheat germ agglutinin (WGA) for chitin, concanavalin A (ConA) for mannans and anti-β-1,3/anti-β-1,6-glucan antibodies for β-1,3/β-1,6-glucans. Binding specificity of these biomolecules were validated using penta-*N*-acetylchitopentaose, α-mannans, laminarin (short β-1,3-glucan chain) and gentiobiose (2 glucose units linked in β 1→6) immobilized on epoxy glass slides. Dynamic force spectroscopy was employed to obtain kinetic and thermodynamic information on the intermolecular interaction of the binary complexes using the model of Friddle-Noy-de Yoreo. Using this model, transition state distance x_t_, dissociate rate k_off_ and the lowest force (*f_eq_*) required to break the intermolecular bond of the complexes were approximated. These functionalized dendritips were then used to probe the yeast cell surface treated with a bacterial protease. As expected, this treatment, which removed the outer layer of the cell wall, gave accessibility to the inner layer composed of β-glucans. Likewise, bud scars were nicely localized using AFM dendritip bearing the WGA probe. To conclude, these functionalized AFM dendritips constitute a new toolbox that can be used to investigate cell surface structure and organization in response to a wide arrays of cultures and process conditions.

## Highlights

• Functionalisation of AFM dendritips with conA, WGA and anti-β-1,3/β-1, 6-glucan antibodies.
• Cell wall polysaccharides were immobilized on epoxy-activated glass slides.
• Specific binding of immobilized polysaccharides to functionalized dendritips.
• Functionalized dendritips used as a new tool to probe yeast cell surface.

## 1 Introduction

The yeast cell wall of *Saccharomyces cerevisiae* is a solid armour of about 150 nm thick, which represents 20–25% of the cell dry mass (Aguilar-Uscanga and Francois, 2003). This cell wall consists of mannoproteins (40–45% of wall dry mass), β-1,3 and β-1,6-glucans (45–55% of wall dry mass) and a tiny amount of chitin (less than 5% of wall dry mass), which are cross-linked in various ways to form higher-order complexes (Klis et al., 2006). Under transmission electron microscopy, the cell wall appears as the juxtaposition of two layers, an outer layer of about 30–40 nm thick that is merely composed of mannoproteins and an inner layer of about 70–100 nm mainly composed of β-glucans (Osumi et al., 1984). These two layers are however interconnected through different covalent linkages of mannoproteins to β-glucans (see Orlean (2012), for a review on the subject). Although the chemical composition of the yeast cell wall is well known, its molecular and spatial organization is still challenging to draw. Moreover, the amount, the physical structure and the interlinkages between cell wall components are subject to dramatic changes in response to various stress, culture conditions, fermentation processes and downstream processing (Aguilar-Uscanga and Francois, 2003, Pillet et al., 2014, Schiavone et al., 2016, Schiavone et al., 2015). The cell wall composition was also reported to be substantially different among yeast species (Nguyen et al., 1998).

Each of the cell wall components is endowed with technological properties that are pertinent for industrial and medical applications. A well-recognized property of the yeast cell wall mannoproteins resides in the adhesion to inert surface and in the consequent formation of biofilms (Blankenship and Mitchell, 2006, Bojsen et al., 2012), as well as in the capacity to interact with aromatic and phenolic compounds during winemaking process (Chalier et al., 2007, Mekoue Nguela et al., 2016, Pradelles et al., 2008). The *manno*-oligosaccharides that decorate cell wall proteins are also reported to exhibit prebiotic properties as illustrated by the stimulation of gut microbiota of chickens (Pourabedin et al., 2017, Pourabedin and Zhao, 2015) and by preventing expansion of pathogenic bacteria via direct connexion to specific fimbriae present at the bacteria surface (Ganner and Schatzmayr, 2012). Moreover, yeast mannans have been reported to improve immune responses and growth efficiency of pigs infected with virus (Che et al., 2012). On the other hand, immuno-stimulatory, anti-tumor and anti-diabetes properties have been documented for β -glucans, which is due to their interactions with receptors among which dectin-1 and complement receptor CR3 are the most characterized (Aleem, 2013, Clemons et al., 2014, Du et al., 2014, Legentil et al., 2015). However, the effects of β-glucans are dependent on the degree of branching and on the enchainment of the glucosyl units (1 → 3, 1 → 4, 1 → 6) (Bohn and Bemiller, 1995, Raa, 2015), what can explain some discrepancies in the reported effects of the polysaccharides originated from various biological sources or isolated by different procedures (Cleary et al., 1999, Kulicke et al., 1997). Chitin also shows immunostimulating properties in mammals (Lee et al., 2008), which are strongly influenced by the size of chitin particles (Cuesta et al., 2003). This polysaccharide was also reported to induce cytokine production for leukocyte recruitment and activation of macrophages (Da Silva et al., 2009, Esteban et al., 2001).

The rapid development of Atomic Force Microscopy (AFM) in liquid has allowed to address several problems on the structural organization and interaction of cell surface with its environment, which could not be attempted by other techniques. The remarkable properties of AFM is to be a non-destructive technique that requires a minimal sample preparation. It enables imaging individual cells at high resolution and measuring interaction forces in the range of piconewtons (Dufrene, 2008, Muller and Dufrene, 2008). Using this tool, quantitative data regarding nanomechanical properties (*i.e.* stiffness, elasticity) of microbial cells have been obtained (Dague et al., 2010, Dague et al., 2007). More remarkably, the functionalization of AFM tips with specific biomolecules or cells opened a new angle of investigating the interaction at the single molecular level (single-molecule force spectroscopy or SMFS) (Alsteens et al., 2011, Hinterdorfer and Dufrene, 2006) or single cell level (single-cell force spectroscopy or SCFS) (Alsteens et al., 2013, Benoit et al., 2000, El-Kirat-Chatel and Beaussart, 2018). Dynamic-force spectroscopy (DFS) can then be employed to measure binding properties of biomolecules in a dynamic manner, which means the force it takes to manipulate a biomolecule or a biomolecular complex (Merkel et al., 1999, Takeuchi et al., 2006). It requires the application of an external force that increases linearly with time so that the molecule or complex is exposed to a constant loading rate. Evans and Ritchie (1997) found that the bond strength, which corresponds to the rupture force between two interacting molecules, varies as the logarithm of the loading rate. However, force spectra are rarely linear, and biological entities such as pili or polymers have shown a nonlinear force response (Bjornham and Andersson, 2017). The nonlinear trend of the force spectra was investigated by Friddle et al. (2012). They proposed a new DFS model based on the existence of two regimes that account for this non-linearity, namely (i) a near equilibrium regime characterized by a finite force *f_eq_* that corresponds to the lowest force required to break the bond and (ii) a kinetic regime characterized by a dependence of the force to the logarithm of the loading rate. Application of this model provides the energy landscape of molecular interaction giving rise to force, dissociate rate and energy barrier of these interactions.

The purpose of this work was to investigate the dynamics of interaction between AFM tips functionalized with biomolecules that shall specifically interact with the four different components of the yeast cell wall. To this end, dendrimer-activated tips (so-called dendritip) were functionalized with either wheat germ agglutinin (WGA), concanavalin A (Con A), anti-β-1,3-glucan or anti-β-1,6-glucan antibodies. We validated the specificity of these functionalized dendritips by probing epoxy-activated glass slides coated with laminarin (a β-1,3-glucan oligosaccharide), gentiobiose (a disaccharide linked in β 1 → 6), penta-*N*-acetylchitopentaose (5 units of *N*-acetylglucosamine linked in β 1 → 4) (see Table 1) and α-mannans as sugar substrates that are representative of the yeast cell wall polysaccharides. Moreover, kinetic and thermodynamic information about these interactions were extracted using the Friddle-Noy-de Yoero model (Friddle et al., 2012). Finally, we employed these functionalized AFM dendritips to explore the yeast cell surface after a protease treatment as well as to map chitin at surface of the yeast cell concomitantly with bud scars.

**Table 1:** Structure of the polysaccharides used in this study.

Name	MW (kDa)	Formula	Chemical representation
Laminarin	∼5	β-1,3-Glucan with ramifications of β-1,6-glucan	
Gentiobiose	0.342	2 units of glucose linked in β(1 → 6)	
Mannan	54	Mannose units linked in α (1 → 4); α (1 → 2); α (1 → 6)	
Penta-*N*-acetylchitopentaose	∼1.0	5 units of *N*-acetyl-d-glucosamine linked in β(1 → 4)	

## 2 Materials and methods

### 2.1 Chemicals and biochemicals

Ethanolamine hydrochloride, tetrahydrofuran (THF), dichloromethane, dimethylsulfoxide (DMSO), sodium cyanoborohydride, acetone, ethanol, bovine serum albumin (BSA), *o*-phenylenediamine (OPD), laminarin from *Laminaria digitate*, α-mannan from *Saccharomyces cerevisiae* (M75014), d-glucose, d-mannose, *N*-acetyl-d-glucosamine were purchased form Sigma-Aldrich and used without further purification. Pustulan (β-1,6-glucan) and β-1,6-gentiobiose were obtained from Calbiochem and Carbosynth, respectively. β-1,3-laminaribiose and penta-*N*-acetylchitopentaose were provided by Megazyme. PBS buffer (10 mM Na_2_HPO_4_, 138 mM NaCl, 2.7 mM KCl, pH 7.4) was purchased in Euromedex. Epoxide-activated glass slides were from Schott Nexterion (Nexterion® Slide E). The MLCT AFM tips were obtained from Bruker. Wheat Germ Agglutinin (WGA) and concanavalin A (ConA) from Sigma-Aldrich. Anti-β-1,3-glucan, a mouse monoclonal IgG was provided by Biosupplies (http://www.biosupplies.com.au/, Australia). The anti-β-1,6-glucan antibody, a rabbit polyclonal antibody IgG, was kindly provided by Prof. Frans Klis (Swammerdam University of Amsterdam). A goat-anti-rabbit and a rabbit anti-mouse IgG-peroxidase conjugates were purchased from ThermoFisher Scientific.

### 2.2 Yeast, culture condition and protease treatment

The yeast *Saccharomyces cerevisiae* wild-type BY4741 (MATa his3Δ1; leu2Δ0; met15Δ0; ura3Δ0) stored at −80 °C was revivified on YPD agar plate and then cultivated in YPD medium (1% w/v yeast extract, 2% w/v peptone, 2% w/v glucose) at 30 °C with shaking at 200 rpm until OD_600_ = 1 (∼1.4 × 10^7^ cells/ml). Unless otherwise stated, cells were harvested by centrifugation (3000 rpm × 3 min), washed with PBS or acetate buffer (18 mM, pH 5.2) and re-suspended to obtain around 5 OD unit at 600 nm. Yeast cells were immobilized by mechanical trapping into polydimethylsiloxane (PDMS) stamps (Formosa et al., 2015a). Firstly, a bare AFM tip was used to visualise and image a single cell before to change it with a functionalized dendritip. For protease treatment, YPD-cultivated yeast cells were treated with 2 mg/ml of *Streptomyces griseus* protease (P6911, Sigma-Aldrich) at 37 °C for 6 h at pH 7.5, then collected by centrifugation and processed as above. Eight cells from 3 independent experiments were analysed for each treatment and a total of 8192 force curves were analysed.

### 2.3 Characterization of antibodies specific for β-1,6-glucan and β-1,3-glucan

The binding specificity of the antibodies was determined by indirect ELISA assay against β-1,3-glucan-BSA and β-1,6-glucan-BSA as described by Montijn et al. (1994). β-1,3-laminaribiose and β-1,6-gentiobiose were conjugated to BSA by reductive amination described by Roy et al. (1984). BSA (6.8 mg, 0.1 µmol), oligosaccharides (10 mg, 29.2 µmol) and NaBH_3_CN (18 mg, 0.159 mmol) were dissolved in 2.5 ml of sodium borate buffer (0.2 M, pH 9.0). The reaction was carried out at room temperature for 96 h with continuous stirring. Conjugates were purified from reaction mixtures using a spin column with a 10-kDa cut off (Amicon, Millipore), lyophilized and resuspended in PBS to a final concentration of 1 mg.ml^−1^. For the indirect ELISA assays, 96-wells plates were coated with either β-1, 3-glucan-BSA or β-1, 6-glucan-BSA (100 µl of 10 µg.ml^−1^ in PBS, pH 7.2). The coated plates were washed four times with PBS (200 µl per well) and incubated for 1 h at 37 °C with serial dilutions (1:5000 to 1:20,000) of purified antibodies diluted in PBS containing 3% (w/v) BSA. The plates were washed four times with PBS, and then incubated with either a goat anti-rabbit or a rabbit anti-mouse IgG-peroxidase conjugate (200 µl per well of dilutions 1:1000 and1:2000) during 1 h at 37 °C. After incubation, the plates were washed 4 times with 100 µl of PBS. Then, 100 µl of a 0.5 mg.ml^−1^ *O*-phenylenediamine solution in peroxidase buffer was added per well as chromogenic substrate. The plates were incubated at 37 °C for 30 min and then read at 450 nm. Control assay was performed by the same procedure with 100 µl of PBS buffer instead of BSA conjugates.

### 2.4 Functionalization of AFM dendritips

The Si_3_N_4_ AFM tips were first functionalized with dendrimers, bearing 96 aldehydes groups according to the procedure described in Jauvert et al. (2012) to give rise to AFM dendritips. Then these tips were incubated for 1 h at room temperature in 100 µl of 0.2 mg.ml^−1^ of lectin solution (ConA or WGA) in 100 mM carbonate sodium buffer pH 8.4 to which 2 µl of 1 M NaBH_3_CN in 100 mM sodium hydroxide was added. For coupling with antibodies, AFM dendritips were incubated overnight at 4 °C in 100 µl of 0.1 mg.ml^−1^ of antibodies solution in 100 mM carbonate buffer with 2 µl of 1 M NaBH_3_CN. After incubation, the AFM dendritips were washed two times in PBS-Tween (PBS containing 0.05% Tween® 20). They were immediately used after functionalization.

### 2.5 Immobilization of polysaccharides on epoxy-activated glass slides

To epoxy-activated glass slides (Nexterion® Slide E from Schott, Germany), 100 µl of 10 mg.ml^−1^ of the different substrates (Table 1) prepared, unless otherwise stated, in PBS buffer was deposited and incubated for 16 h at room temperature. As the coating is sensible to light, the slides were kept in dark. After incubation, the slides were washed two times with 100 µl PBS. For ConA experiment, substrates were prepared in 18 mM sodium acetate buffer, pH 5.2 containing 1 mM MnCl_2_ and 1 mM CaCl_2_ and the slides were washed with the same buffer.

### 2.6 Single molecular atomic force spectroscopy measurements

The force spectroscopy experiments were carried out with a Nanowizard III atomic force microscope (Bruker-JPK Instruments). The spring constant of each dendritip was determined by the thermal noise method (Hutter and Bechhoefer, 1993) and were found to be in the range of 10–20 pN/nm. All experiments with antibodies and WGA were performed in PBS buffer. Experiments using ConA-AFM tips were done in sodium acetate buffer (18 mM, pH 5.2) containing 1 mM CaCl_2_ and 1 mM MnCl_2_. AFM images were recorded in the Quantitative Imaging mode (Chopinet et al., 2013), at 20 °C in buffer solution. Force–distance curves were recorded in buffer solution at different retraction speeds ranging from 1 to 6 µm.s^−1^ and at a constant applied force of 500 pN. Adhesion force maps were obtained by recording 32 × 32 force–distance curves. For specific blocking of the interaction, 100 µl of 0.2 M solution of d-glucose, d-mannose or *N*-acetyl-d-glucosamine was added to the embedded cells in the PDMS chamber before AFM measurements

### 2.7 Dynamic force spectroscopy

To determine the parameters of energy landscapes for each couple of interacting molecules the values of rupture forces were determined from the force-distance curves recorded at different loading rates. Thousands of approach-retract cycles were carried out to provide sufficient data to fit to the theoretical model. The intermolecular interactions of biomolecules were described as free energy landscapes using the Friddle-Noy-de Yoreo model (Friddle et al., 2012). In this model, the rupture force ***F*** that varies as a function of the loading rate r can be approximated by the following equation:

(1) $$F\left(r\right)≅f_{\mathrm{eq}}+f_{\beta }*ln\left(1+\frac{re^{-\gamma }}{f_{\beta }*koff\left(f_{\mathrm{eq}}\right)}\right)$$

where *f_eq_* is the equilibrium force, f_β_ is the thermal force, γ is the Euler’s constant (γ = 0.577) and K_off_ is the dissociate rate constant at *f_eq_*. The loading rate r is defined as the change in force with time, therefore it influences the rupture force and bond survival.

The thermal force f_β_ is defined as:

(2) $$f_{\beta }=\frac{k_{B}T}{x_{t}}$$

where k_B_ is the Boltzmann’s constant, T is the temperature and x_t_ is the width of the energy barrier.

The equilibrium force *f_eq_* is defined as the force at which the dissociation and association rate intersect and is given by the equation:

(3) $$f_{\mathrm{eq}}=\sqrt{2k_{c}\Delta G}$$

where k_c_ is the spring constant of the cantilever and ΔG is the Gibbs free energy of binding, representing the height of the activation barrier.

The bond lifetime is calculated as

(4) $$\tau =1/k_{\mathrm{off}}$$

Origin 8.0 software (OriginLab) was used to fit Eq. (1) to experimental DFS plot with a three-parameters nonlinear fit, along with 99% confidence intervals and 99% prediction intervals of the rupture forces. Fitting convergence criteria were approximated with a nonlinear iterative Levenberg-Marquardt algorithm. The maximum of iterations were fixed at 500, the chi-square (χ^2^) tolerance between each iteration was fixed at 10^−15^. The parameters *f_eq_*, *k_off_*, *f* β were extracted from fitting and parameters x_β_ and ΔG were calculated using Eqs. (2), (3).

### 2.8 Data analyses

AFM images and force-distance curves were analysed with the JPK Data Processing software (Bruker-JPK Instruments).

## 3 Results and discussion

### 3.1 Functionalization of AFM dendritips with wheat germ agglutinin, concanavalin A, anti-β-1,3 and anti β-1,6-glucan antibodies

To address the probing of the different polysaccharides that compose the yeast cell wall at a nanoscale level, we functionalized AFM tips with a biomolecule or ligand that should unambiguously recognize these cell wall macromolecular components. Until now, AFM tips bearing concanavalin A (ConA) that specifically interacts with mannnosyl units of *manno*-oligosaccharides have been used to map mannoproteins at the yeast surface (Alsteens et al., 2008, Beaussart et al., 2012, Formosa et al., 2015b, Francius et al., 2009, Schiavone et al., 2015). El-Kirat-Chatel et al. reported the functionalization of AFM tips with wheat germ agglutinin (WGA) and monoclonal anti-β-1,3-glucan antibody to probe chitin and β-glucans, respectively, at the cell surface of different yeast species and after a heat shock treatment (El-Kirat-Chatel et al., 2013). The use of WGA to detect chitin was justified by early works showing that this lectin possesses eight adjacent sugar-binding sites (Schwefel et al., 2010), which binds to GlcNAc or β-(1,4)-linked oligomer derivatives, as determined by extensive studies using NMR, fluorescence and X-Ray diffraction experiments (Kristiansen et al., 1999, Lotan and Sharon, 1973, Wright et al., 1984). In this work, we wished to revisit the specificity and accuracy of the lectins and antibodies that interact with cell wall polysaccharides, starting from the process of chemical functionalization of silicon-nitride (Si_3_N_4_) AFM cantilevers with dendrimers to the immobilization of polysaccharide substrates on epoxy-activated glass slides.

We first verified the specificity of anti β-1,3- and anti β-1,6-glucans antibodies by performing ELISA assays against BSA-β-(1,3)-glucan and BSA-β-(1,6)-glucan conjugates according to the procedure of Montijn et al. (1994). Indirect ELISA titration of polyclonal anti-β-1,6-glucan antibodies against BSA-gentiobiose, a two units of glucose linked by a β 1 → 6 bond (Table 1), showed a half saturation value of 206 ng.ml^−1^ with no interference with BSA-β-1,3-laminarin. This result shows the high specificity of this antibody to β-1,6-glucan since laminarin from *laminari digitila* used in this work is reported to be a short linear β1,3-glucan chain cross-linked with β-1,6-glucan at a ratio of 7 (Hrmova and Fincher, 1993). On the other hand, a value of 30 ng.ml^−1^ was obtained for the anti-β-1,3-glucan antibody against BSA-laminarin with no cross-reactivity with gentiobiose, in accordance with the data from the commercial supplier (data not shown). In a second step, we fabricated dendritips by amination of the silicon nitride AFM tips with ethanolamine followed by incubation of these amino-derived tips with G4-aldehyde dendrimers as described in Jauvert et al. (2012). These AFM dendritips were afterwards incubated with either WGA, ConA, anti-β-1,3-glucan or anti- β −1,6-glucan antibodies. This incubation was followed by reduction of the imine bond formed between the dendrimers and the silicon nitride tip and between dendrimers and the epsilon amino group of lysine of the protein molecules into amine in the presence of sodium cyanoborohydride. The biofunctionalized AFM dendritip were then be used for single-molecule force spectroscopy analyses.

The third step was to qualify the interaction of these functionalized AFM dendritips with their cell wall substrates. To this end, we sought to use epoxy-activated glass slides coated with soluble polysaccharides that are representative of β-1,3-glucan, β-1,6-glucan, chitin and α-mannans. The hydroxyl groups present on the sugar polysaccharides should react with the epoxide function, leading to a covalent linkage between the sugar and the glass slide. We validated this coating strategy by spotting laminarin – a β-1,3-glucan substrate, penta-*N*-acetylchitopentaose, a substrate derived from chitin, and purified yeast α -mannans on epoxy-activated glass slides (Table 1). The coating of the first two substrates, which were biotinylated prior spotting on the glass slide was verified with streptavidin–Alexa fluor®647, whereas ConA labelled with this fluorophore was used to show that α-mannans were immobilized on the epoxy-activated glass slide. In addition, we showed that only WGA-Alexa fluor®647 binds to the immobilized chitopentaose (see Figs. S1 and S2 in Supplementary data).

### 3.2 The functionalized AFM dendritips specifically recognize their cognate substrates immobilized on epoxy-activated slides.

**Fig. 1:**
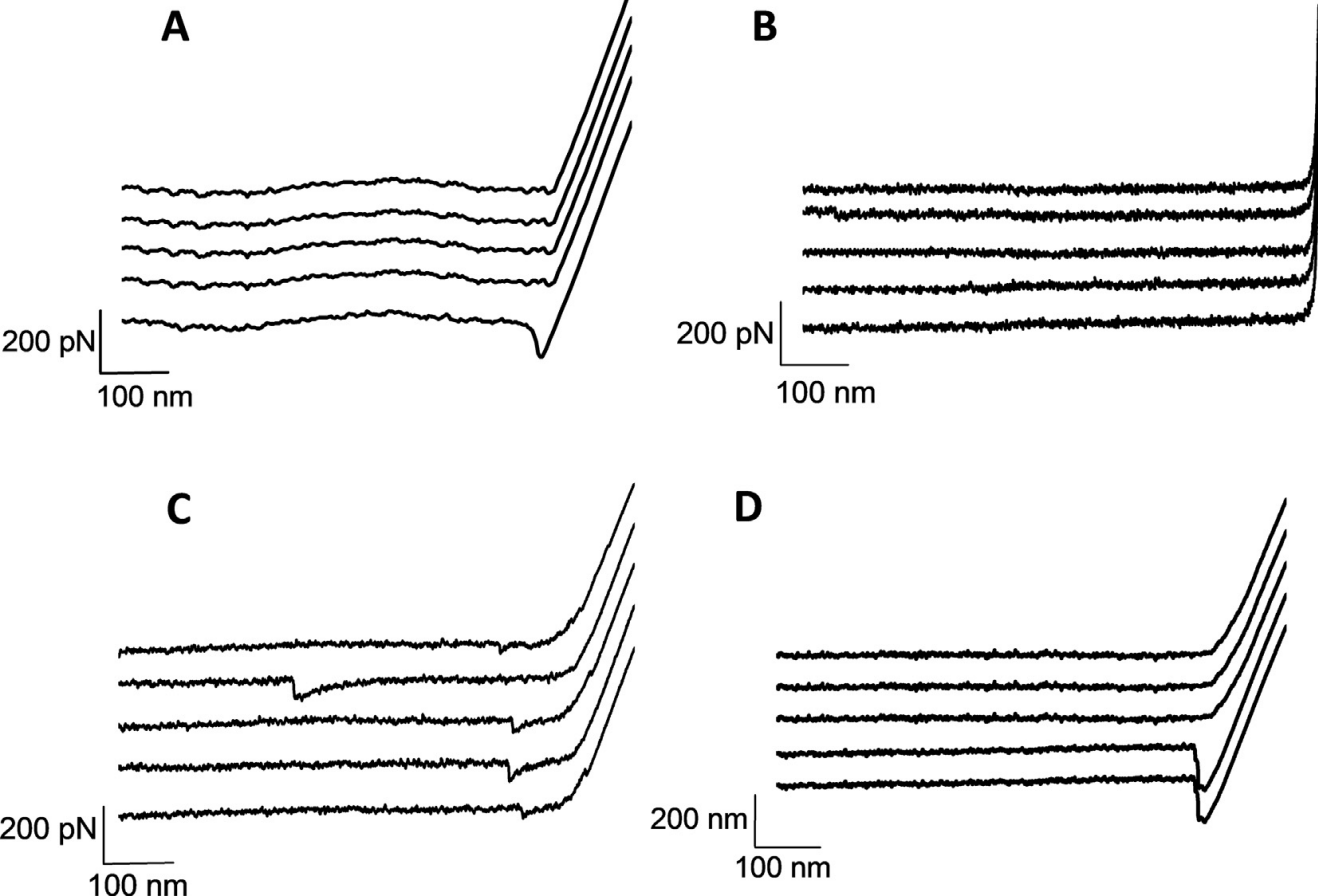
Specificity of the interaction of AFM dendritips functionalized with anti-β-1,3-glucan antibodies on epoxide-activated glass slide alone (A) or coated with laminarin (C) and gentobiose (D). The interaction of AFM tips is reported as force-distance curve, at a retraction rate of 1 µm.s^−1^. In (B) is shown the result with a naked AFM dendritip on laminarin-coated slide.

**Fig. 2:**
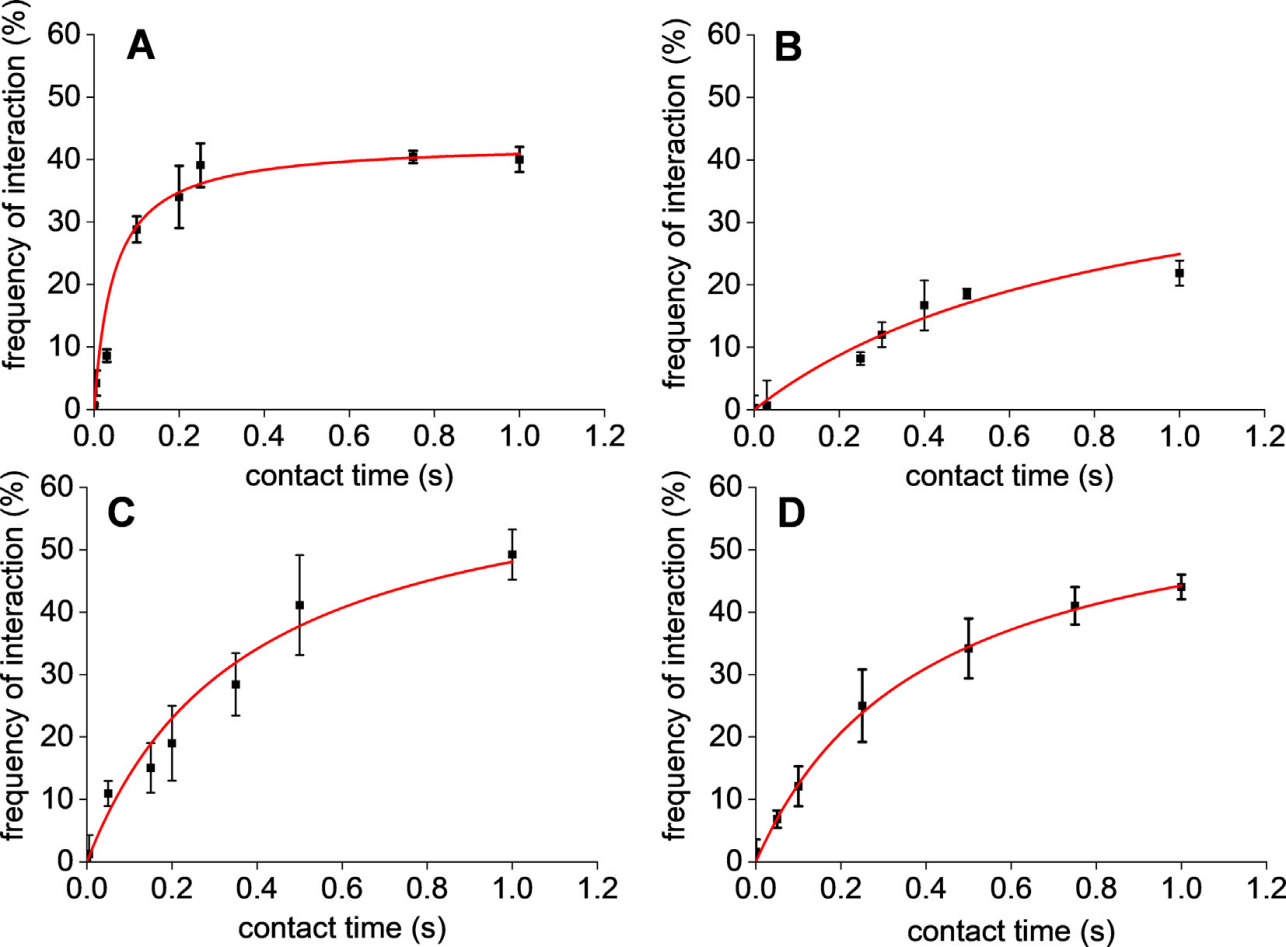
Effect of the contact time on the interaction between functionalized AFM dendritips and their cognate substrates immobilized on epoxy-activated glass slide. (A) interaction of monoclonal anti-β-1,3-glucan antibody to laminarin-coated slide; (B) interaction of polyclonal anti-β-1,6-glucan antibody to gentiobiose-coated slide, (C) interaction of WGA to penta-*N*-acetylchitopentaose-coated slide and (D) interaction of conA to α - mannan-coated slide.

When probing the surface of a pristine glass slide with a AFM dendritip bearing anti-β-1,3-glucan antibodies, a deflection peak at the contact point with the surface was obtained (Fig. 1A). Similar data were obtained with AFM dendritips functionalized with the other biomolecules, such as with WGA (Fig. S3). Also, we did not find any interaction between AFM dendritip functionalized with anti-β-1,3-glucan antibodies and epoxy-activated glass slide coated with gentiobiose (Fig. 1D). Likewise, no interaction of WGA-tip to pristine glass slides or coated with laminarin was found (Fig. S3). In contrast, deflections at distance ranging from 40 to 500 nm were recorded from the retraction of an AFM dendritip functionalized with anti-β-1,3-glucan antibody on a laminarin-coated glass slide (Fig. 1C), demonstrating the specificity of the interaction of this antibody to its substrate. To further validate the specificity and affinity of the functionalized AFM dendritips towards their cognate polysaccharide substrates, we carried out time course interaction experiments at a constant applied force of 500 pN. Results of this experiment showed that the kinetic of the interaction frequency for each of the functionalized AFM dendritips to their polysaccharide target followed a seemingly hyperbolic curve that can be fitted to a Michaelis-Menten model (Fig. 2). Plotting the values according to a double reciprocal plot, *i.e.* 1/frequency of interaction vs 1/contact time allowed us to estimate a half saturation value (k_0.5_) for each couple of interacting complex. This k_0,5_ could express the time needed to reach 50% of the maximal interaction frequency between the functionalized AFM dendritip and its immobilized polysaccharide target, under the conditions specified in Methods. As shown in Fig. 2, the interaction of the AFM dendritip bearing anti-β-1,3-glucan antibodies and laminarin was the most faster, giving rise to a k_0.5_ 10–20 fold lower than that of interaction between dendritip functionalized with WGA, con A or anti-β-1,6-glucan and its corresponding polysaccharides target (Table S1).

### 3.3 Force spectroscopy analyses for molecular interaction and determination of unbinding force

**Fig. 3:**
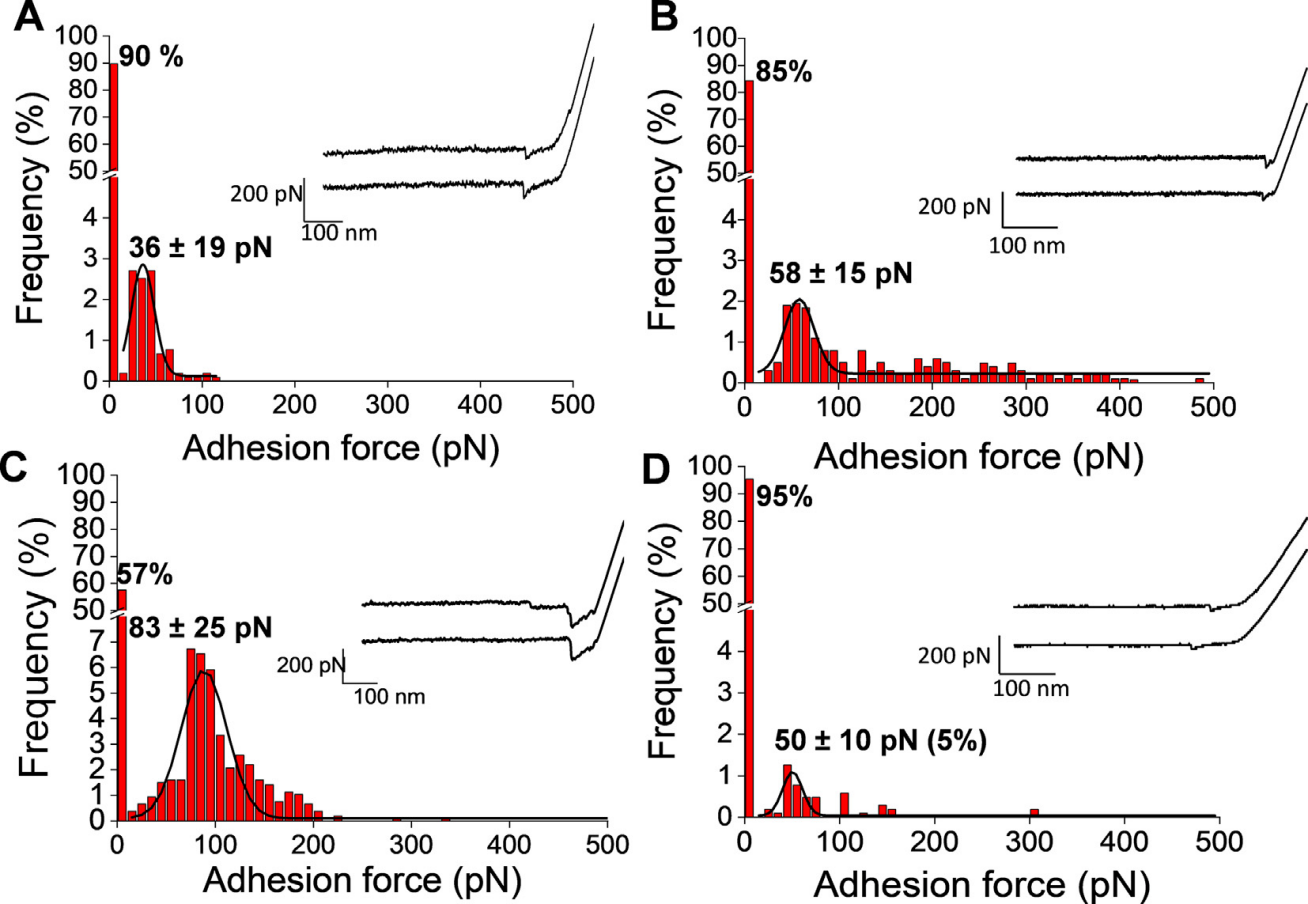
Determination of unbinding force and frequency of adhesion of functionalized AFM dendritips to their specific substrate coated on epoxy-activated glass slide coated. The frequency, the estimated unbinding force and force-curve distance are shown with AFM tip bearing in (A) monoclonal anti-β-1,3-glucan antibody versus laminarin, in (B) polyclonal anti-β-1,6-glucan antibody vs gentiobiose, in (C) WGA vs penta-*N*-acetylchitopentaose and in (D) conA vs mannan.

By collecting thousands of force-distance curves at a retraction speed of 1 µm.s^−1^ between the immobilized polysaccharide substrates (target) and the AFM dendritips functionalized with their specific probes *ie*. l.ectins or antibodies, we could draw a distribution of the adhesion force versus the frequency of the interaction for each probe/ target couple (Fig. 3). The representation of these force-distance curves revealed that essentially single adhesion events were recorded, which was spread over a rupture distance ranging from 20 to 200 nm. Also, the adhesion frequency of WGA to the penta-*N*-acetylchitopentaose was significantly higher than that with the other probes to their target (from 30 to 51% vs 5 to 15%), arguing that the association constant (k_on_) of penta-*N*-acetylchitopentaose substrate to WGA probe is stronger than that of the other probes-polysaccharides substrates. By fitting these distributions to a Gaussian curve, a mean adhesion (or unbinding) force was estimated at 36 ± 19 pN for the interaction between anti- β-1,3-glucan and laminarin, and 83 ± 25 pN for WGA/chitopentaose at a pulling rate of 1 µm.s^−1^. These data were comparable to those reported by El-Kirat-Chatel et al. (2013)) using heat-treated yeast cell. Indeed, these authors identified two maximum forces at 42 ± 7 pN and 84 ± 24 pN when probing the surface of the heat-treated cell with an AFM tip bearing a monoclonal anti-β-1,3-glucan antibody. While the value of the first peak is similar to ours, the second peak at 84 pN may be explained by the interaction with two β-glucan chains. The same authors also reported an adhesion force of 54 pN for the interaction of chitin with WGA while a force of 83 pN was estimated under our condition (Fig. 3). This difference may be attributed to the number of GlcNAc units of the chitin chains that can interact with WGA, since it has been reported that the strength of the WGA-GlcNAc interaction is proportional to the number of GlcNAc units (Bains et al., 1992). We also found that the adhesion force of 50 ± 10 pN we determined between ConA-functionalized AFM tip and α-mannan immobilized on an epoxy-activated glass side was comparable to that reported by Francius et al. (2009) (*i.e.* 57 ± 19 pN) for a single ConA-mannose interaction. Finally, we report for the first time an unbinding force of 58 ± 15 pN that characterized the interaction of anti-β-1,6-glucan antibodies functionalized AFM dendritip to gentiobiose immobilized on epoxy-glass slide.

### 3.4 Energy landscape of the intermolecular interactions between cell wall polysaccharides and their specific probe determined by dynamic force spectroscopy

**Fig. 4:**
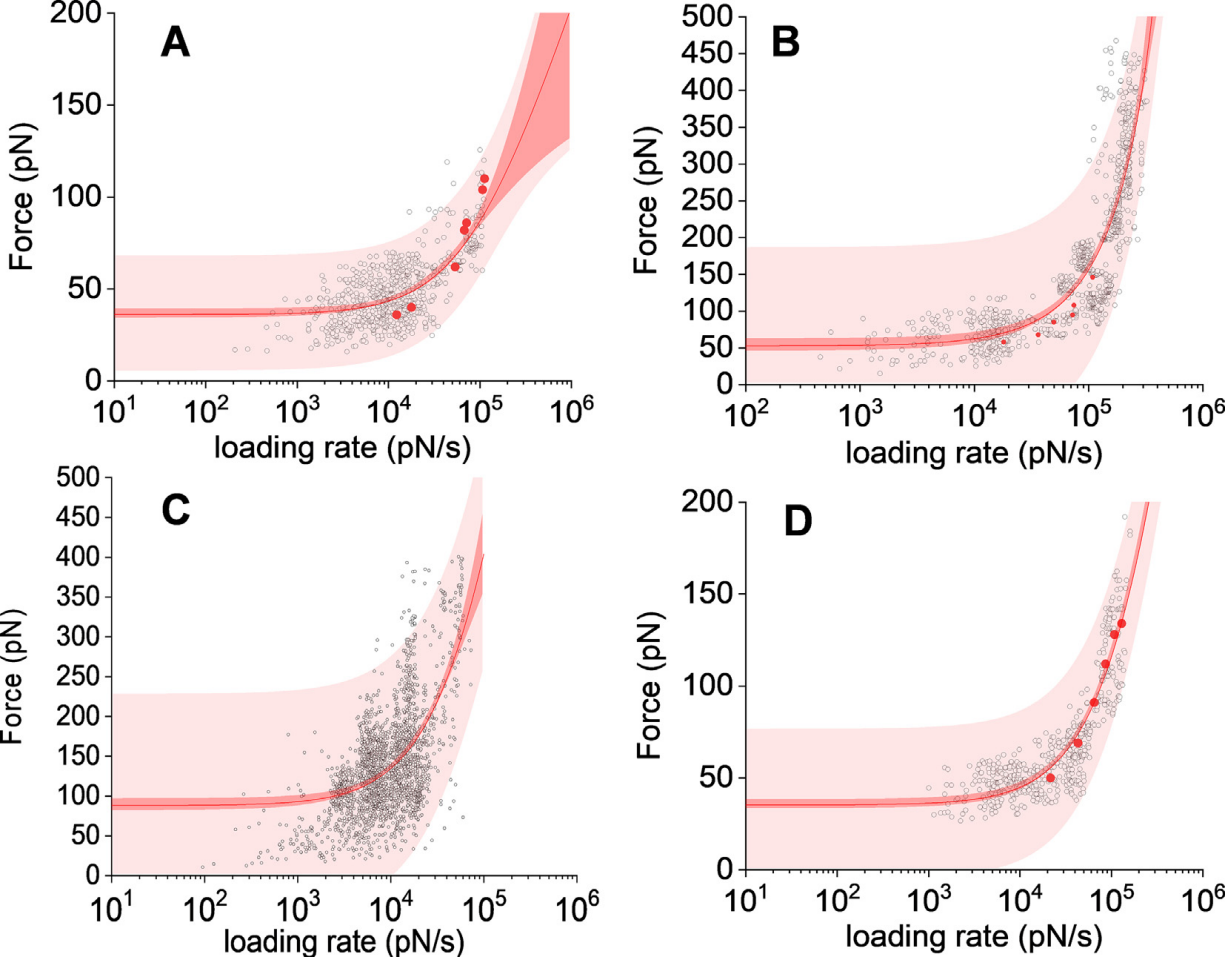
Dynamic force spectra of functionalized AFM dendritips towards their specific substrates immobilized on epoxide-activated glass slides. The figure reports the rupture force vs the logarithm of the loading rate of an AFM dendritip functionalized with (A) anti-β-1,3-glucan antibody probing a laminarin-coated slide, (B) anti-β-1,6-glucan antibody probing gentiobiose-coated slide, (C) WGA probing a penta-*N*-acetylchitopentaose-coated slide, and (D) conA probing α-mannan-coated slide.

We next exploited DFS to explore the energy landscape of the intermolecular bonds that characterize the interactions between each of the biomolecules covalently fixed on the AFM dendritips and their corresponding polysaccharides target immobilized on epoxy-activated glass slide. According to the theory of Evans (2001), the probability to rupture a molecular bond increases with increasing force at a constant loading rate. However, and in contrast to Evans’ law, a non-linear behavior in the force versus loading rate spectra was obtained on a range of loading rate from 100 to 20,000 pN/s. This behavior is illustrated in Fig. 4 where each dot corresponds to the critical loading rate, which is the loading rate at the exact rupture of the interactions (see also Figs. S4–S7, which illustrate the frequency of interaction and the peak force calculated at different loading rate for each couple of probe-polysaccharides substrates). These nonlinear trends are typical of intermolecular interactions between biological entities and have been modelled by Friddle et al. (2012). Indeed, to account for this nonlinearity, the model proposed that the force spectrum reflects two fundamental regimes of bond rupture: (i) a near equilibrium regime between rupture and reforming bond, independently of the loading rate, and (ii) a kinetic regime characterized by a dependence of the rupture force to the log- loading rate (f ∼ ln r). Fitting the analytical approximation of the Friddle equation to the dynamic force vs loading rate spectra for each couple of interacting biomolecules within a confidence interval of 99% (Fig. 4), kinetic and thermodynamic parameters of the non-covalent intermolecular bonds between the polysaccharide and their specific probe could be approximated (Table 2). The *f_eq_*, corresponding to the lowest force required to break the bond ranged from 35 to 88 pN, which agrees with those commonly obtained for single-bond interaction (Evans, 2001, Friddle et al., 2012). These *f_eq_* values were actually close to the adhesion force reported in Fig. 3. With these data and using Eq. (3) (defined in Material and methods), the free energy barrier ΔG needed to create the bonding complex could be calculated. This ΔG for the WGA/chitopentaose was 10 times higher than that for the binding energy of this complex obtained by isothermal titration calorimetry (Bains et al., 1992), whereas it was 2 times lower than that determined by QCM (Coulibaly and Youan, 2014) for the ConA/α-mannans complex. These discrepancies can be due to difference in pH, temperature and solvation between the assay conditions, which are known to affect the free energy of binding of these ligands. The model can give access to the transition state distance *x_t_*_,_ which corresponds to the width of the energy barrier. The calculated values were lower than 1 Å, which is in the range of most of the biomolecular complex interactions (Friddle et al., 2012, Lv et al., 2012). This low distance also indicated that the bond cannot withstand a large deformation when a high force is exerted. We also determined the *k_off_* that characterizes the dissociate rate constant of the intermolecular bonds of the complex according to Eq. (1) (In Material and methods section). This value also gave access to the lifetime of the bonds (τ_0_ = 1/*k*_off_). As indicated in Table 2, the *k*_off_ of WGA/N-acetylchitopentaose complex was 5–8 times lower than that estimated for the 3 other biological complexes (anti-β-1,3-glucan antibodies/laminarin, anti-β-1,6-antibody/gentiobiose and conA/α-mannan). These data indicated that the intermolecular bonds between WGA and chitin substrate are stronger than those of the other biological complexes, which is further supported by a ΔG that is also higher for the formation of the WGA/penta-*N*-acetylchitopentaose complex (Table 2). To conclude, our dynamic force spectroscopy analysis showed that the intermolecular interactions of cell wall polysaccharides with their specific probes fall into a single-barrier (single bond) model from which the transition state distance *x_t_*, unbinding rate *k*_off_ and equilibrium *f_eq_* could be determined.

**Table 2:** Fitted parameters of the energy landscape for the interaction of functionalized AFM dendritips with specific probe to cell wall polysaccharides. The Friddle-Noy-de Yoreo model (Friddle et al., 2012) has been used to determine the energy landscape of the intermolecular interaction between each couple of polysaccharide with its specific probes covalently attached on a AFM dendritip. Details about the nature of each of the parameters is described in the Results section.

Functionalized AFM dendritip	Polysaccharides coated on epoxide-glass slide	X_t_ (Å)	f_eq_ (pN)	k_off_ (s^−1^)	τ_0_ (ms)	ΔG (kcal.mol^−1^)
Anti-β-1,3-glucan antibodies	β-1,3-Laminarin	0.6 ± 0.2	36.2 ± 1.0	732.4 ± 10.4	1.37	−5.3
Anti-β-1,6-glucan antibodies	β-1,6-Gentiobiose	0.3 ± 0.1	52.7 ± 4.1	1046.7 ± 197.6	0.96	−11.0
Concanavalin A	α-Mannan	0.2 ± 0.1	35.3 ± 1.0	569.9 ± 15	1.75	−4.2
Wheat germ agglutinin	β-1,4-Penta-*N*-acetylchitopentaose	0.1 ± 0.04	88.1 ± 2.9	113.6 ± 10.1	8.81	−55.1

### 3.5 Applications of biofunctionalized AFM dendritips to probe surface of living yeast cells after a proteolytic treatment and during budding.

**Fig. 5:**
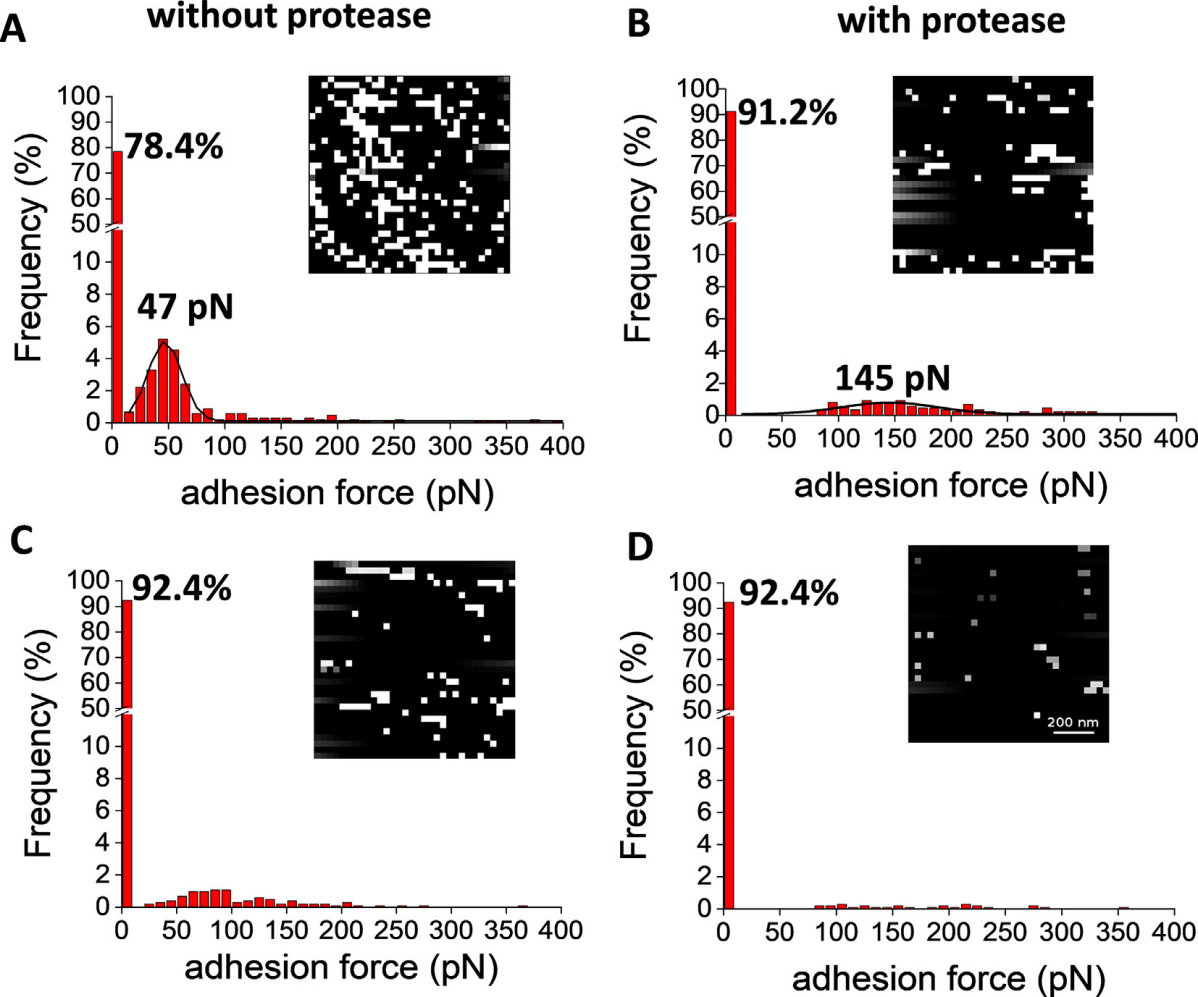
Molecular mapping of a yeast cell surface using AFM dendritip functionalized with ConA before (A, C) and after (B, D) protease treatment. Inserts are represented the adhesion force map, where white pixels correspond to adhesion events whereas black pixels represent absence of interaction. In (C, D) are shown adhesion force after addition of excess of mannnose to the immobilized cells.

We used our functionalized AFM dendritips to probe the surface of yeast cells prior and after a treatment with a bacterial protease. The rational of this proteolytic treatment was to remove the outer layer of the cell wall made of mannoproteins to get access to the inner layer composed of β -glucans, as originally reported by Zlotnik et al. (1984). As shown in Fig. 5, the adhesion events recorded by probing the untreated yeast cell with a ConA-functionalized AFM dendritip dropped to less than 8% after addition of 200 mM d-mannose, suggesting that the remaining interactions were likely nonspecific. In addition, the mean adhesion force (47 pN) measured for this interaction was comparable to the force of a single ConA-mannose interactions (Francius et al., 2009). Upon digestion of the yeast cell wall with the protease, the frequency of adhesion events dropped to a level similar to that after addition of mannose to untreated yeast cell, arguing that the mannoproteins coat of the cell wall has been largely removed by this treatment (Fig. 5B). Addition of excess of mannose prior to AFM interaction resulted in the loss of minor binding events exhibiting adhesion forces in the range of 100–300 pN (Fig. 5D). However, about 8% of unspecific interactions as observed with untreated cells remained.

**Fig. 6:**
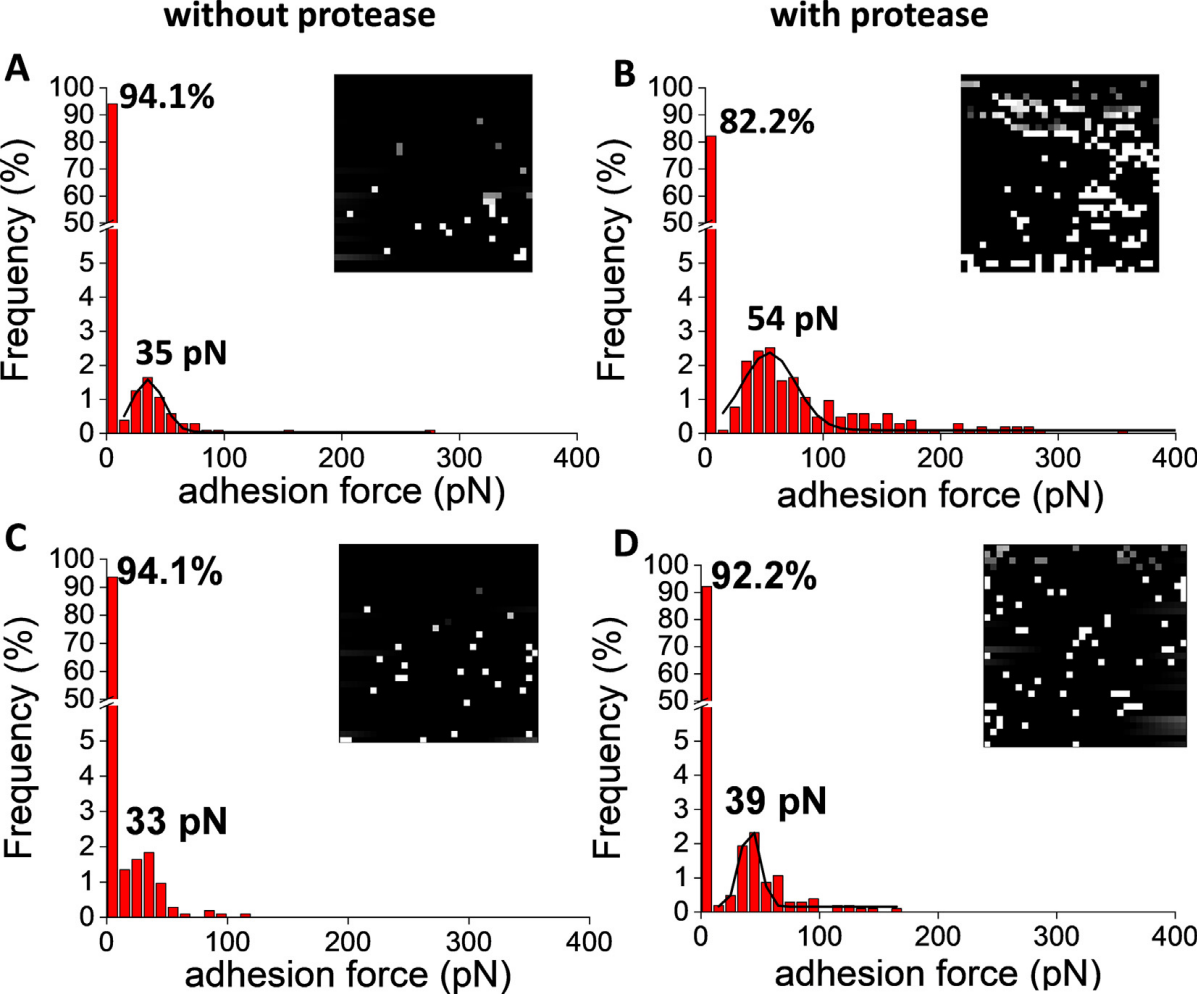
Molecular mapping of yeast cell surface using an AFM dendritip functionalized with anti-β-1,3-glucan (A, B) and anti-β-1,6-glucan antibodies (C, D) before (A, C) and after (B, D) protease treatment. AFM tip functionalized with either anti-β-1,6-glucan antibody on a yeast cell before (C) and after protease treatment (D). Force maps and histograms of the frequency of adhesion of the tip functionalized on the yeast cell surface in function of the adhesion force detected.

We next used an AFM dendritip functionalized with anti-β-1,3-glucan antibodies to probe the cell surface before and after the protease treatment (Fig. 6). As expected, the interaction events of an untreated yeast cell with this functionalized dendritip were almost inexistent as shown also on the adhesion force map (<6%, Fig. 6A), even though there were some minor and most likely unspecific interaction events that were spread in the range of 10 to 100 pN of adhesion force. In contrast, the frequency of adhesion events significantly increased by almost 20% by probing the protease-treated cell with this functionalized dendritip (Fig. 6B). This result confirmed that the cell wall outer layer has been removed and has given access to the inner layer composed of β-1,3-glucan. However, the mean adhesion force peaked at ∼54 pN instead of 36 pN as determined earlier using laminarin-coated glass slide. As already noticed above, the binding affinity of anti β-1,3-antibody to β-glucan has been reported to be dependent on its molecular mass, degree of branching and structure (Mueller et al., 2000, Strandh et al., 1998). Clearly the molecular size and structure of yeast β-1,3-glucan chains are different from the laminarin which is a short β-glucan chain containing glucose mostly linked in β 1 → 3 (Table 1). When probing the cell surface with AFM dendritip functionalized with an anti-β-1,6-glucan antibody, again very few detection events were recorded on the untreated yeast cell (Fig. 6C). However, the frequency of the interaction slightly increased with the protease-treated yeast cells (Fig. 6D). Since this weak increase of interaction frequency with a mean adhesion force peaking at 39 pN was abolished when excess of glucose was added prior to AFM experiment (data not shown), we could conclude that β-1,6-glucans chains were recognized on the yeast surface after treatment with the protease.

**Fig. 7:**
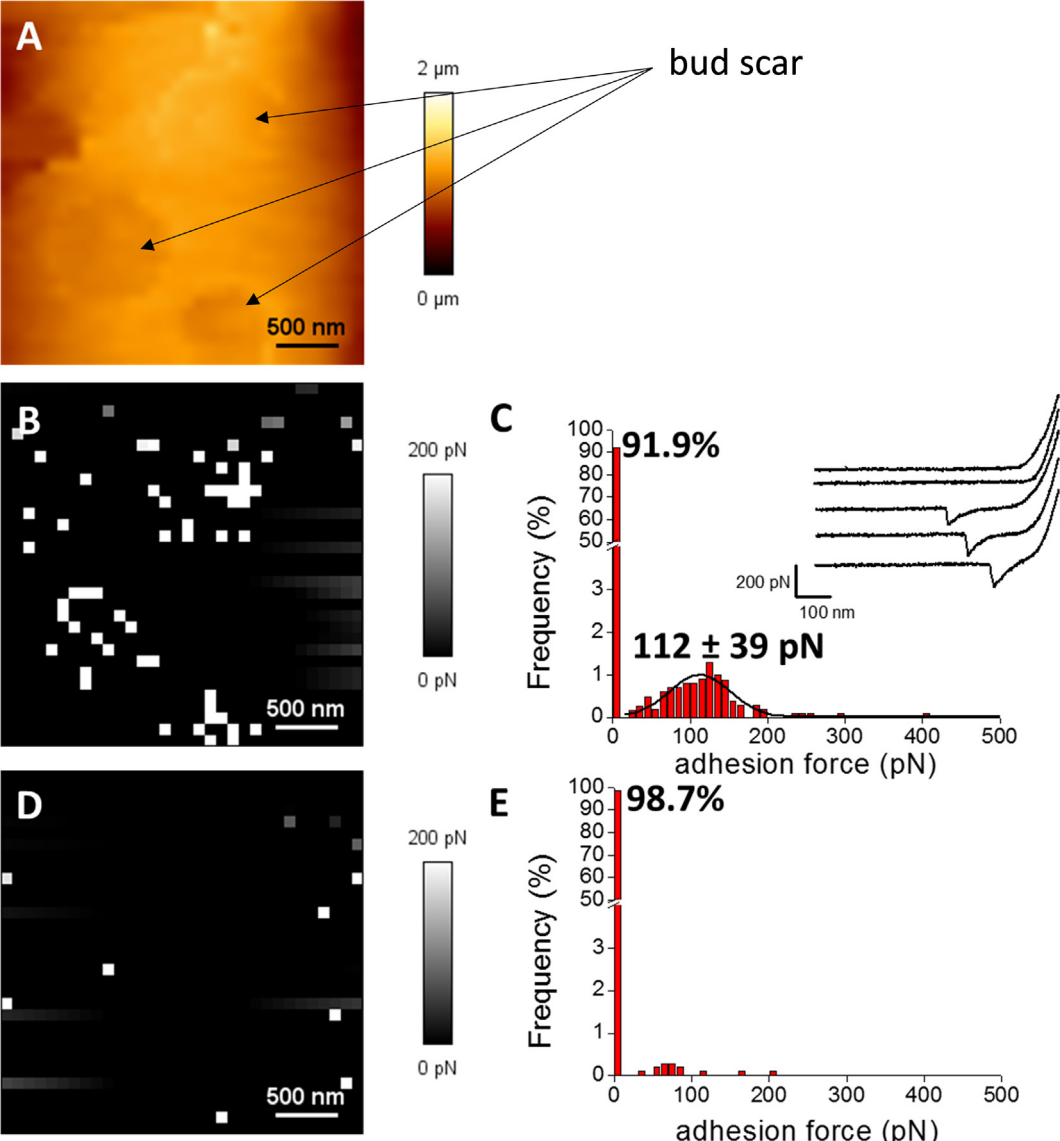
Molecular mapping of cell surface using an AFM tip functionalized with wheat germ agglutinin. In A is shown the AFM image at high resolution from a single cell of the yeast *S. cerevisiae*, illustrating the presence of 3 bud scars. Panel B & C correspond to the adhesion force maps recorded in PBS buffer between the AFM dendritip functionalized with WGA and the yeast cell surface in the absence (B) or after addition of 200 mM *N*-acetyl glucosamine (C). White pixels indicate specific adhesion events, whereas black pixels represent non-adhesive events. Corresponding adhesion force histograms (for 1024 force –distance curves) of C and E are shown in panel D and F.

We finally evaluated the pertinence of our AFM dendritip functionalized with WGA by searching the cell surface for bud scars, as they are reported to be highly enriched in chitin (Cabib and Arroyo, 2013). We first employed a simple AFM tip to image a yeast cell, which enabled to localize three bud scars on the surface (Fig. 7B). Using the AFM dendritip bearing WGA as the probe, we mapped the same region and recorded 1024 force -distance curves. We showed that the adhesion force maps overlapped with the localization of the bud scars, which is consistent with the high concentration of chitin in this region (Fig. 7C). In addition, the distribution of the adhesion force ranged from 20 to 200 pN with a maximum at 112 pN. This value is higher than that previously reported (54 pN in El-Kirat-Chatel et al. (2013) and 83 pN, see Fig. 3), which can be explained by the number of GlcNAc units interacting with WGA. Finally, we confirmed that the WGA-functionalized AFM dendritip specifically interacted with *N*-acetylglucosamine moieties of chitin since this interaction was lost upon addition of excess (200 mM) of *N*-acetylglucosamine prior to another force mapping measurements (Fig. 7E & F). Taking these values, we can approximate that the binding of WGA with one GlcNAc unit is approximatively 27 pN.

## 4 Conclusions

In this paper, we report on the development and use of AFM dendritips functionalized with anti-β-1,3-glucan and anti-β-1,6-glucan antibodies, wheat germ agglutinin and concanavalin A to probe β-1,3-glucans, β-1,6-glucans, α-mannans and chitin that compose the yeast cell wall. We qualified these functionalized AFM dendritips by determining their specificity towards their cognate cell wall polysaccharides previously immobilized on epoxy glass slides. We then demonstrated that intermolecular interactions between cell wall polysaccharides and their specific molecular probes fall into the single-barrier or single-bond model proposed by Friddle et al. (2012). Using this model, we could extrapolated the transition state distance x_t_, the dissociate rate k_off_ and the lowest force (*f_eq_*) that is required to break the intermolecular bond of each of the complexes. We then applied these functionalized AFM dendritips to probe the cell surface of yeast before and after a protease treatment, which was aimed at removing the outer-mannoproteins-layer. Accordingly, the frequency of interaction of the surface of the treated cells with an AFM tip bearing ConA dropped to near zero while the adhesion events with an AFM dendritip functionalized with anti-β-1,3-glucan significantly increased. Finally, using the AFM dendritip coated with WGA, we showed that the adhesion force maps nicely overlapped with bud scars, consistent with abundant level of chitin in these specific structures of the cell wall. Altogether, we report in this paper a new AFM-associated toolbox that can be applied to explore cell surface of any fungal cell made of mannoproteins, glucans and chitin.

## Funding

This work was supported in part by a grant from Lallemand Inc. (project Lallwall, n°SAIC2016/048 & SAIC/2018/010).

## Declaration of Competing Interest

The authors declare that they have no known competing financial interests or personal relationships that could have appeared to influence the work reported in this paper.
