# Supplementary material for: AFM dendritips functionalized with molecular probes specific to cell wall polysaccharides as a tool to investigate cell surface structure and organization
Source: Cell Surf. 2019 Aug 5;5:100027. doi: 10.1016/j.tcsw.2019.100027 (PMC7389267; doi:10.1016/j.tcsw.2019.100027)

Table S1: Relative 'affinity' and maximal frequency of interaction of AFM dendritips functionalized with probes that specifically interact with polysaccharides substrates immobilized on epoxide-activated glass slides

Functionalized AFM tips	Polysaccharides coated on the epoxide glass slide	$K_{0.5}$ (s)*	Maximal interaction (%)*
Anti β-1,3-glucan antibodies	β-1,3-laminarin	0.046 ± 0.19	42.8 ± 2.1
Anti β-1,6-glucan antibodies	β-1,6-gentobiose	0.86 ± 0.48	46.5 ± 16.5
Concanavalin A	α-mannan	0.40 ± 0.04	61.9 ± 2.3
Wheat germ agglutinin	β-1,4-Penta-N-acetylchitopentaose	0.37 ± 0.10	66.1± 8.7

The values were determined using Lineweaver–Burk representation (double reciprocal plot [($\frac{1}{V} = \frac{Km}{Vmax}.\frac{1}{S} + \frac{1}{vmax}$] of the fitting data according to a Michaelis-Menten model in Figure 2. V = frequency of the interaction of the AFM tips with the sugars on the glass slide; S = contact time

Figure S1. Spotting of biotinylated laminarin on epoxy-activated glass slides (Nexterion® SlideE from Schott) (a) or nitrocellulose membrane (Nitrocellulose Fast® Slides 16 pads format from Schleicher & Schuell) (b) and detection by streptavidine-Alexa Fluor® 647.

Qarray min from Genetix (U.K) was employed to spot solution of biotinylated laminarin at the indicated concentration prepared in PBS 1X buffer. After the spotting, the slides were dried overnight. The interaction with the labelled target was realized in dark for 30 min to 1 h at 25°C in PBS1X. After washing two times the slides with PBS1 containing 0.005% Tween20, the slide were read on AXON 4000B at 100 % PMT.

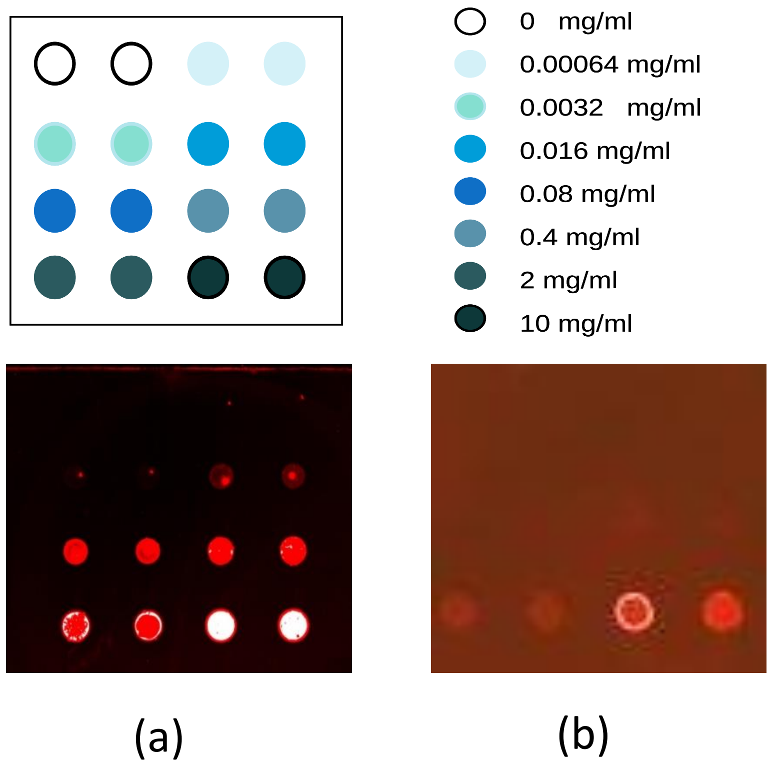

Figure S2: Spotting of biotinylated N-acetylchitopentaose on epoxy activated -glass side and detection with made with (a) streptavidin-Cy3 or (b) WGA_Alexa Fluor®647 and (c) ConA_Alexa Fluor®647 Experimental design as in Fig.S1, except that the buffer for interaction of labelled ConA with α-mannans was PBS 1X containing 1 mM $CaCl_2$ and 1 mM $MnCl_2$.

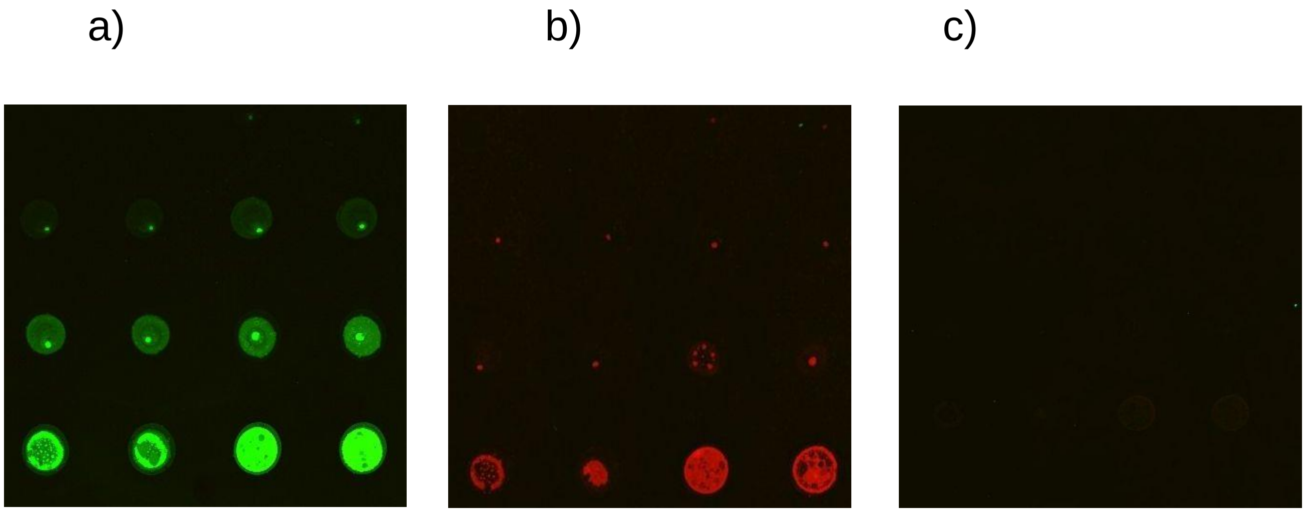

Figure S3. Lack of interaction of AFM dendritips functionalized with WGA on naked epoxy-activated glass slide (A) or coated with laminarin (B). Experiment design as in Fig.1

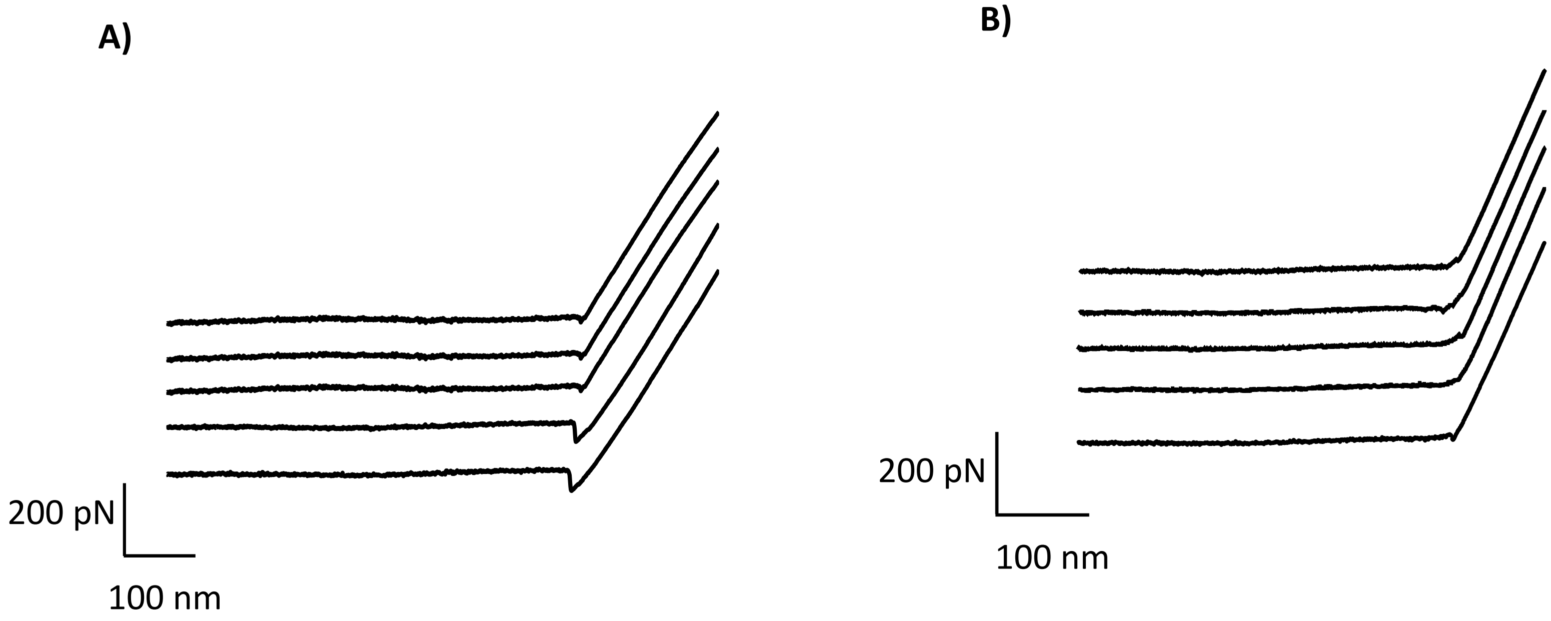

Figure S4. Frequency of interaction as a function of the rupture force recorded from AFM dendritip functionalized with anti-β(1,3)-glucan probing laminarin coated epoxy-activated glass slide at different loading rates.

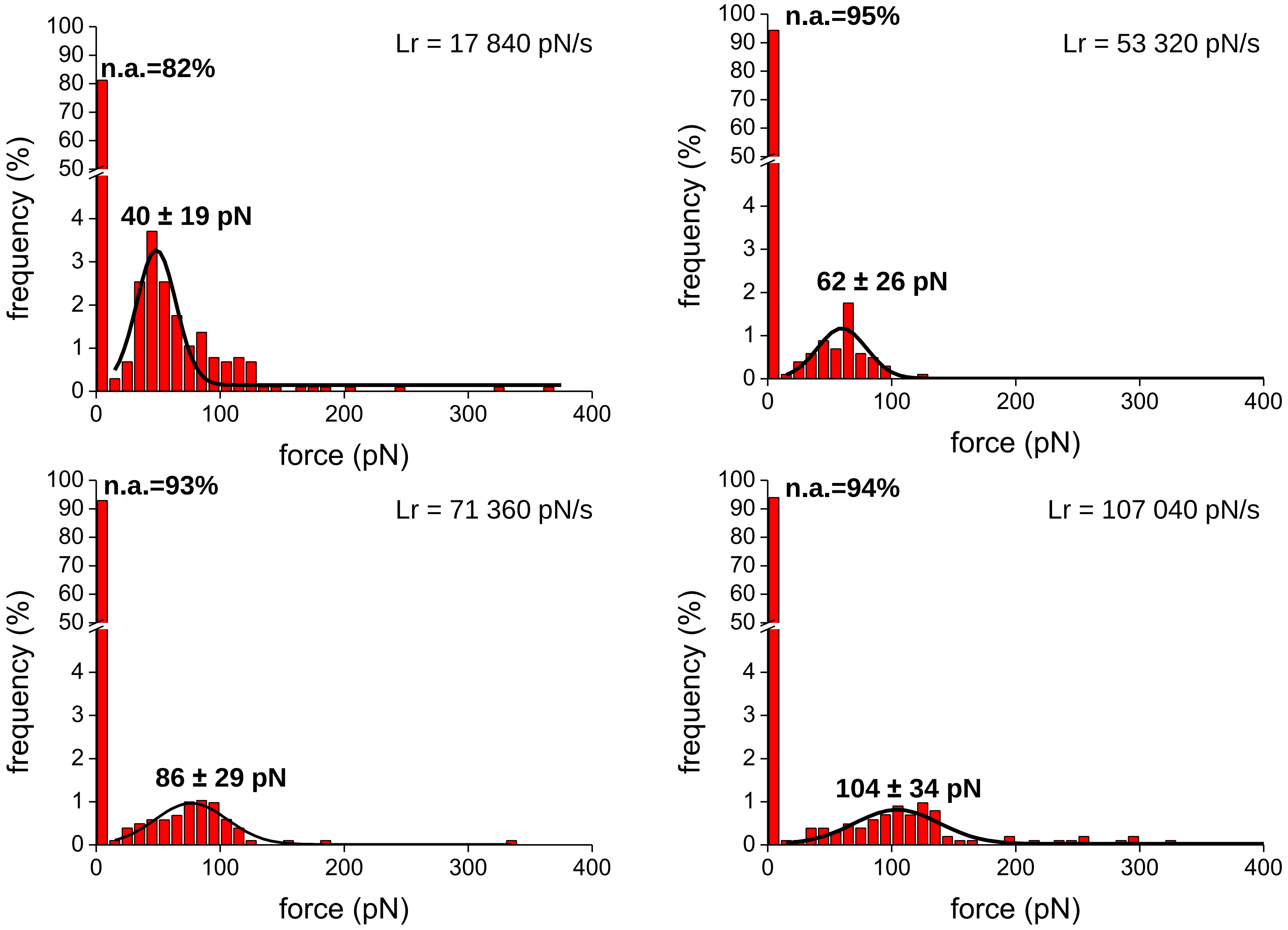

Figure S5. Frequency of interaction as a function of the rupture force recorded from an AFM dendritip functionalized with anti-β(1,6)-glucan probing a gentiobiose coated epoxy-activated glass slide at different loading rates.

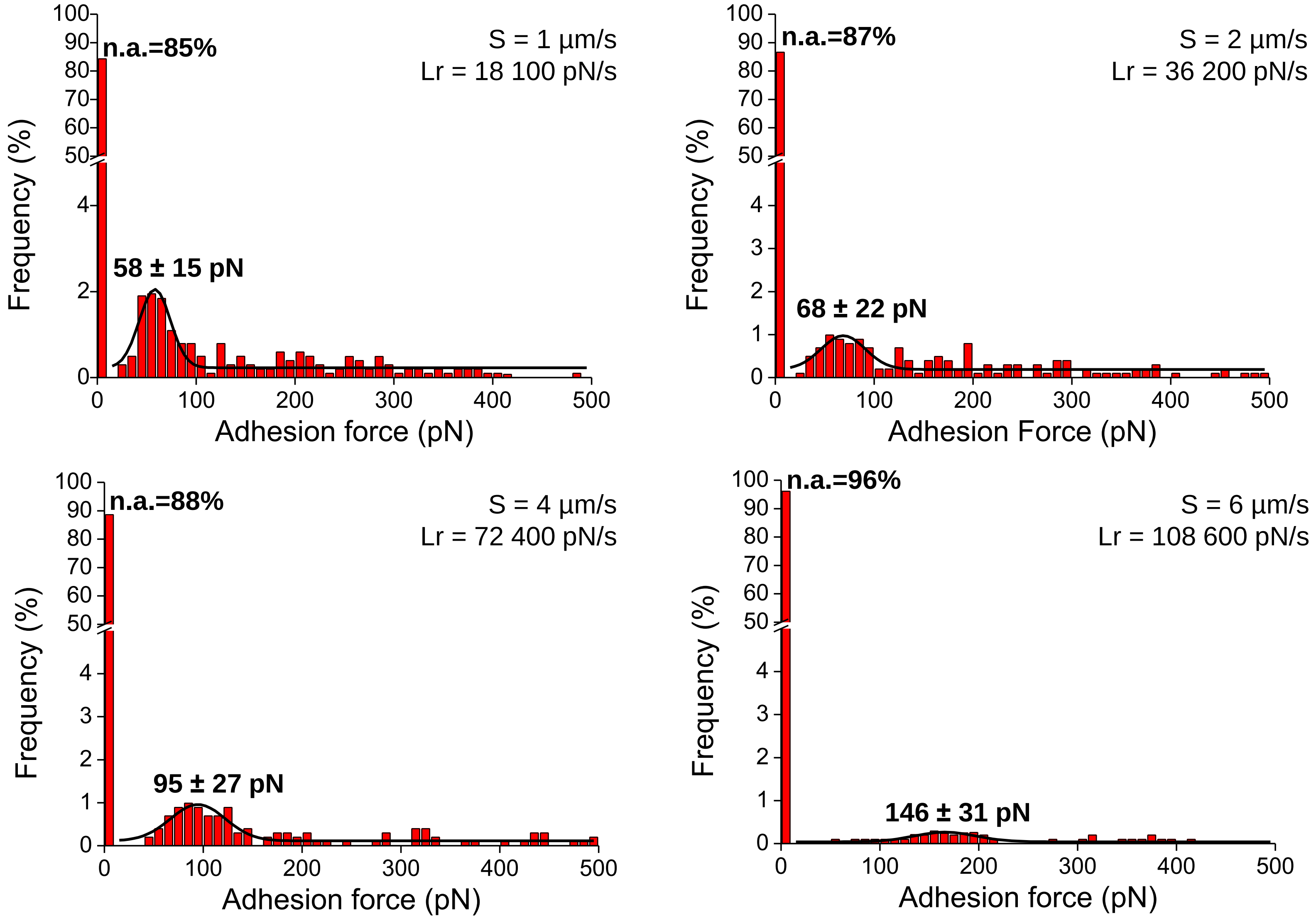

Figure S6. Frequency of interaction as a function of the rupture force recorded from an AFM dendritip functionalized with WGA probing a penta-N-acetyl-D-chitopentaose coated epoxy-activated glass slide at different loading rates.

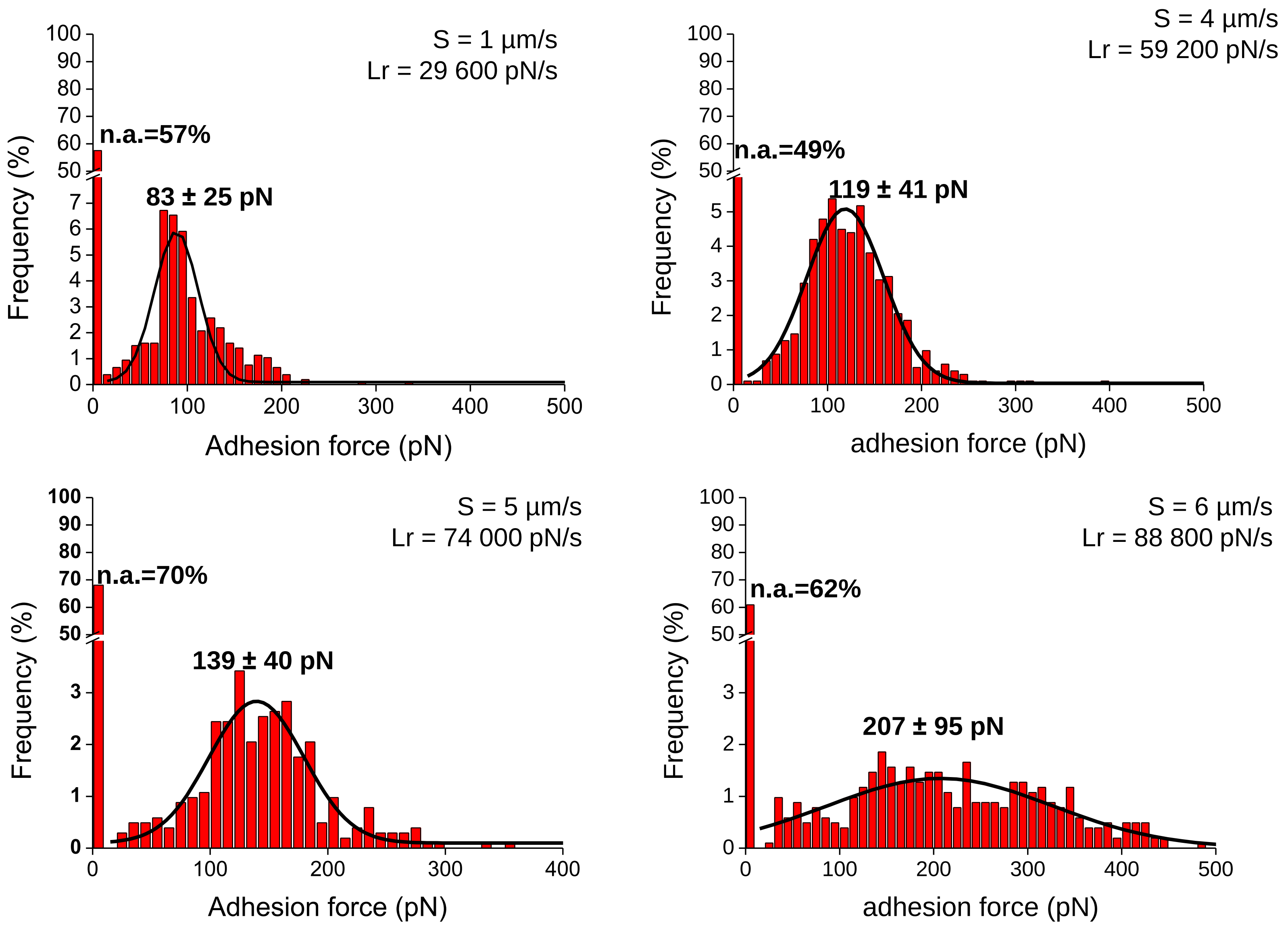

Figure S7. Frequency of interaction as a function of the rupture force recorded from an AFM dendritip functionalized with ConA probing a α- mannan coated epoxy-activated glass slide at different loading rates.

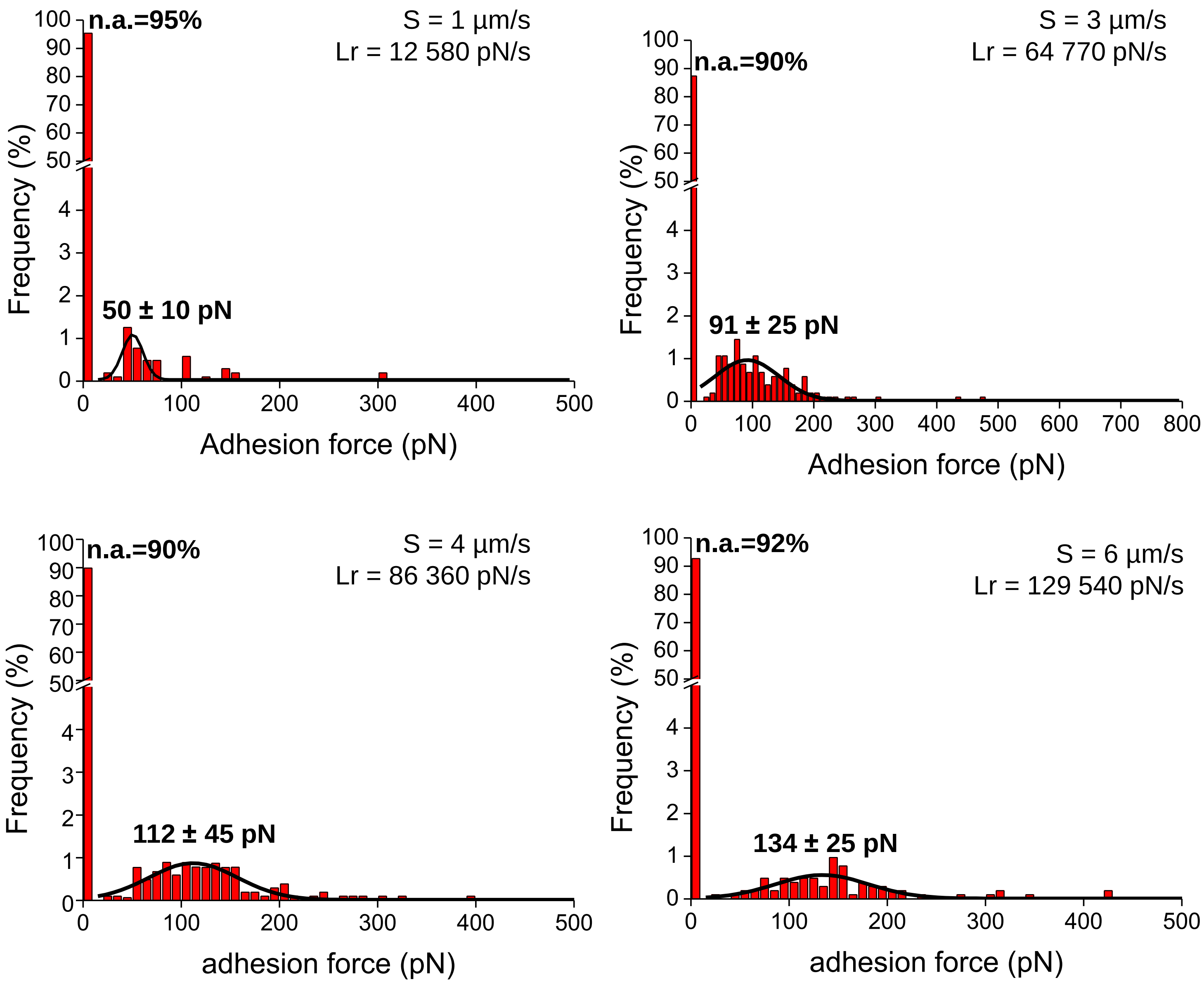

Supplement: Supplementary data 1 [file mmc1.pdf]
